# Diffraction-limited hyperspectral mid-infrared single-pixel microscopy

**DOI:** 10.1038/s41598-022-26718-6

**Published:** 2023-01-06

**Authors:** Alexander Ebner, Paul Gattinger, Ivan Zorin, Lukas Krainer, Christian Rankl, Markus Brandstetter

**Affiliations:** 1 RECENDT — Research Center for Non-Destructive Testing GmbH, 4040 Linz, Austria; 2 Prospective Instruments LK OG, 6850 Dornbirn, Austria

**Keywords:** Applied optics, Mid-infrared photonics

## Abstract

In this contribution, we demonstrate a wide-field hyperspectral mid-infrared (MIR) microscope based on multidimensional single-pixel imaging (SPI). The microscope employs a high brightness MIR supercontinuum source for broadband (1.55 $\upmu \hbox {m}$–4.5 $\upmu \hbox {m}$) sample illumination. Hyperspectral imaging capability is achieved by a single micro-opto-electro-mechanical digital micromirror device (DMD), which provides both spatial and spectral differentiation. For that purpose the operational spectral bandwidth of the DMD was significantly extended into the MIR spectral region. In the presented design, the DMD fulfills two essential tasks. On the one hand, as standard for the SPI approach, the DMD sequentially masks captured scenes enabling diffraction-limited imaging in the tens of millisecond time-regime. On the other hand, the diffraction at the micromirrors leads to dispersion of the projected field and thus allows for wavelength selection without the application of additional dispersive optical elements, such as gratings or prisms. In the experimental part, first of all, the imaging and spectral capabilities of the hyperspectral microscope are characterized. The spatial and spectral resolution is assessed by means of test targets and linear variable filters, respectively. At a wavelength of 4.15 $\upmu \hbox {m}$ a spatial resolution of 4.92 $\upmu \hbox {m}$ is achieved with a native spectral resolution better than 118.1 nm. Further, a post-processing method for drastic enhancement of the spectral resolution is proposed and discussed. The performance of the MIR hyperspectral microsopce is demonstrated for label-free chemical imaging and examination of polymer compounds and red blood cells. The acquisition and reconstruction of Hadamard sampled 64 $\times$ 64 images is achieved in 450 ms and 162 ms, respectively. Thus, combined with an unprecedented intrinsic flexibiliy gained by a tunable field of view and adjustable spatial resolution, the demonstrated design drastically improves the sample throughput in MIR chemical and biomedical imaging.

## Introduction

Infrared (IR) microscopy is a powerful analytical technique for the investigation of chemically and spatially inhomogeneous samples in point-and-shoot scenarios, via scanning spectroscopy approaches, or via hyperspectral imaging at the microscopic level. IR microscopy captures spatially distributed absorption properties of a sample, which—when examined in the mid-infrared (MIR) spectral range—can be directly assigned to absorption bands that correspond to specific chemical bonds. Molecular information can therefore be obtained directly, contactless, label-free, in ambient air and without destroying the sample^1^. Current fields of application include for instance surface, particle, and failure analysis in forensics^2^, materials science^3^, art restoration^4^, pharmaceutical^5^ and polymer industries^6^, as well as cell and tissue imaging in biological sciences^7,8^.

Over decades IR instruments have been based on Fourier-transform infrared (FTIR) spectrometers, equipped with thermal IR sources and single-pixel or array detectors. In contrast to dispersive spectrometers (i.e. mono- and polychromators) FTIR instrumentation benefits from Fellget’s and Jacquinot’s sensitivity advantages and thus partially compensates for the poor spectral brightness provided by thermal emitters^9^. However, due to the insufficient brightness of thermal IR sources, hyperspectral image acquisition using FTIR microscopes is either based on time-consuming scanning approaches, or on imaging with expensive focal plane array (FPA) detectors with a limited number of pixels. In order to overcome the limitations in measurement time and noise performance of FTIR instrumentation, recent developments in IR microscopy employ high-brightness MIR laser sources as emitters, i.e. tunable quantum cascade lasers (QCL), or more recently supercontinuum laser sources (SCL)^10,11^. In contrast to thermal emitters, QCLs provide (i) a spectral brightness increased by a factor of at least ${10^{4}}$ and (ii) monochromatic but spectrally tunable and unidirectional emission. A fast tuning mechanism in combination with the available optical power density enables the direct application of a fast high-resolution IR FPA detector to achieve hyperspectral imaging without the need for a dedicated spectrometer. However, the spectral bandwidth accessible with QCLs is either relatively narrow, or—by combining several QCLs with adjacent spectral ranges—they become very expensive in terms of price per accessible wavelength. In contrast to already well-established QCL technology, which superseded FTIR spectroscopy as the gold standard in numerous applications^12–15^, MIR SCLs represent an attractive and emerging fiber-based technology with unique properties. Since supercontinua are generated in confined structures of single-mode fibers, MIR SCLs offer high spatial coherence and excellent beam quality, which makes them perfectly suitable for diffraction-limited imaging^16–20^. SCLs are typically pumped by pulsed laser systems emitting at telecommunication wavelengths (1.55 $\upmu \hbox {m}$), which then generate supercontinua through multiple non-linear effects in optical fibers. Thus, MIR SCLs emit spectrally broadband pulses, with each pulse spanning wavelength ranges from the visible or near-infrared (NIR) to the MIR up to 18 $\upmu \hbox {m}$^21,22^. For instance, a MIR supercontinuum covering the spectral range from 1.6 $\upmu \hbox {m}$ to 11 $\upmu \hbox {m}$ with a total optical power of 417 mW was reported recently^23^.

In imaging applications, the spectral brightness of MIR laser sources ensures a sufficiently high power density to exceed the noise levels of high-resolution FPAs with decent detectivity. However, whereas such FPAs are low-cost in the visible spectral range, in the MIR (e.g. mercury cadmium telluride (MCT) or micro-bolometer arrays) they are significantly more expensive, often commercially unavailable due to military application regulations, and their technical characteristics lag noticeably behind their single-pixel counterparts. Thus, especially in the MIR spectral range, the single-pixel imaging (SPI) technique represents a cost-effective solution for high-resolution imaging with superior sensitivity.

In SPI, a spatial light modulator (SLM) is applied to mask an image with time-varying patterns before it is sensed by a single-pixel detector. Synchronized intensity measurements for each of the masking patterns enable the reconstruction of the originally projected images by solving the resulting system of linear equations as reported in^24^. By applying a fast SLM such as a digital micromirror device (DMD) with frame rates of up to 22kHz, image acquisition times can reach the scale of tens of milliseconds. The actual acquisition time mainly depends on the desired resolution and can be further reduced by means of compressive sampling approaches—a profound guideline can be found in the relevant literature^25–27^. However, the main advantages of SPI—particularly in the infrared spectral range—are given by (i) the fact that Fellget’s multiplex advantage can be exploited in the spatial domain, and (ii) the ability to use single-pixel detectors with superior detectivity that outperform individual pixels of high-resolution FPAs at (iii) relatively low costs.

Although these benefits have been successfully demonstrated for hyperspectral SPI in the near-infrared (NIR)^28,29^, to the best of our knowledge, in the MIR, only early proof of concept has been reported so far. In 2012, Russell et al. applied an MIR-modified DMD in a Fabry-Perot filter-based hyperspectral imager^30^. With this instrument, first images of a resolution test target illuminated by a broadband thermal MIR source could be reconstructed successfully. On the one hand, this publication perfectly emphasizes the need for commercially available MIR-ready DMDs, which could transfer this technology to numerous potential applications. On the other hand, since only test targets without significant spectral features were examined, the great importance of *hyperspectral* imaging in the MIR spectral range—which, in contrast to the NIR, probes specific chemical bonds—could not be addressed. Furthermore, an expected, meanwhile well-reported and decisive phenomenon, namely the diffraction of MIR radiation at DMD micromirrors of about < 14 $\times$ 14 $\upmu \hbox {m}^2$ ^31–34^, was not considered in the experimental setup.

In this contribution, we present an SPI approach for hyperspectral MIR microscopy with a MIR-modified DMD and a broadband MIR SCL. In contrast to existing literature, we do take diffraction at the DMD micromirrors into account and exploit the DMD not only for spatial coding, but also as dispersive optical element for spectral discrimination. Thereby, we are tackling the aforementioned drawbacks of FTIR *and* QCL microscopy. We demonstrate diffraction-limited MIR hypersepctral image acquisition without a dedicated spectrometer or additional dispersive elements (e.g. gratings) and apertures (or slits). With a presented numerical calibration scheme to correct for distortions in the hyperspectral cube, a native spectral resolution better than 118.1 nm was achieved and an additional calibration method for drastic enhancement of the spectral resolution is proposed and discussed. The sufficiency of both the realized spatial *and* spectral resolution for label-free chemical imaging is illustrated by the hyperspectral imaging of polymer compounds and human blood cells. Hadamard sampled 64 $\times$ 64 images of the respective samples were acquired and reconstructed in 450 ms and 162 ms, respectively. In addition, the demonstrated MIR microscope provides the intrinsic flexibility of a tunable field of view (FoV) and tunable spatial resolution, which directly correlates with the acquisition time and thus drastically improves the sample throughput in MIR chemical and biomedical imaging.

## Results

### MIR hyperspectral single-pixel microscopy

**Figure 1:**
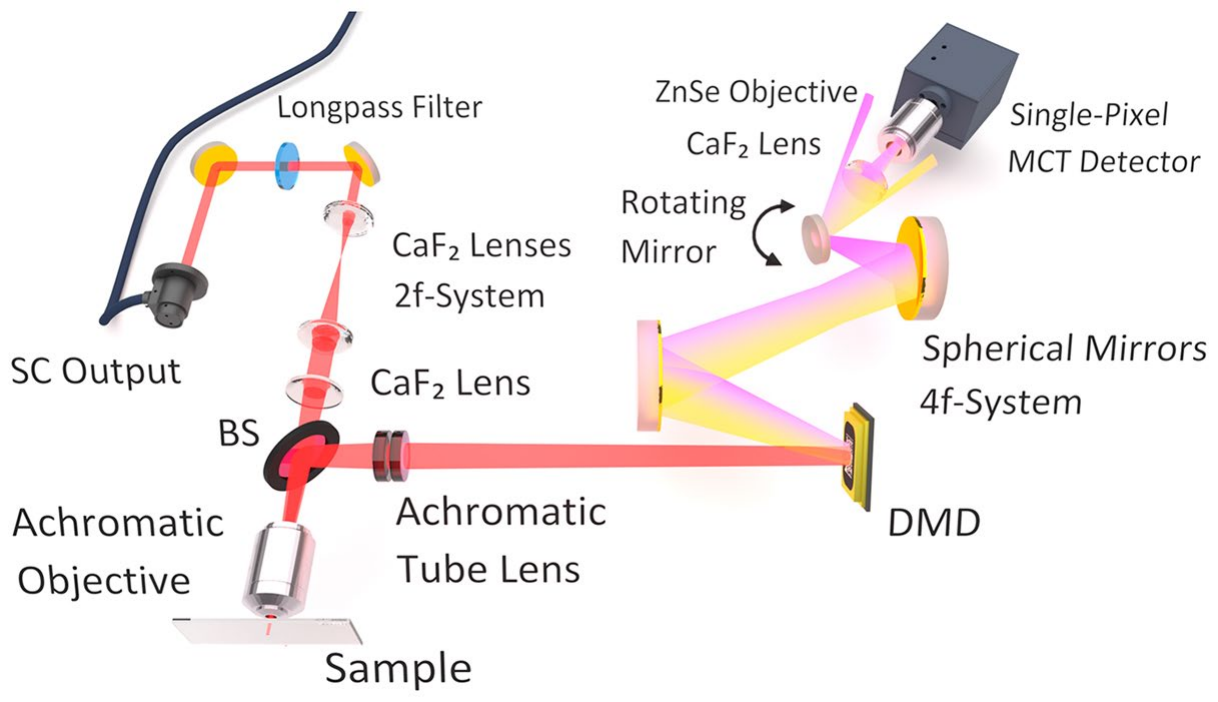
Schematic illustration of the experimental setup. The emission of an MIR SCL passes two uncoated gold mirrors and a 2.4 $\upmu \hbox {m}$ longpass filter before it illuminates the sample by means of a set of three CaF_2_ lenses and an achromatic ZnSe objective. A pellicle beamsplitter (BS) redirects the radiation to an achromatic tube lens, which images the sample onto an MIR-modified DMD. The DMD sequentially masks the projected image and angularly disperses its constituting spectral components due to diffraction. A 4f-system of spherical gold mirrors replicates the DMD field and unifies the spectral images on a rotating gold mirror which selects the spectral band that is projected onto a single-pixel MCT detector by means of a CaF_2_ lens and a ZnSe objective.

The developed hyperspectral MIR SPI microscope, illustrated schematically in Fig. 1, employs a broadband SCL (SuperK MIR, NKT Photonics), which emits in the spectral range from 1.55 $\upmu \hbox {m}$ to 4.5 $\upmu \hbox {m}$ (6450 $\hbox {cm}^{-1}$–2220 $\hbox {cm}^{-1}$) at a repetition rate of 2.5 MHz. The pulsed emission (sub-ns pulse duration) provides a total optical power of 490 mW with 230 mW confined in the spectral range above 2.4 $\upmu \hbox {m}$^19^. For proper alignment, the SCL emission is redirected by means of two adjustable uncoated gold mirrors. In order to avoid overlapping diffraction orders in the detection arm, a MIR longpass filter with a cut-on frequency of 2.4 $\upmu \hbox {m}$ (68-653, Edmund Optics) was implemented in the beam path. Before illuminating the sample, the radiation passes a set of three CaF_2_ lenses of 1 inch diameter followed by an infinity corrected 25x achromatic objective (892-0004, PIKE Technologies) with a numerical aperture (NA) of 0.4. While the first 2f-system of CaF_2_ lenses with focal lengths of 25 mm and 50 mm, respectively, expands the beam diameter by a factor of 2 mm to about 5 mm, the combination of the objective and the third lens with a focal length of 100 mm provides a collimated sample illumination in the given wide-field microscopy configuration. The radiation reflected from the sample is then redirected into the detection unit by means of a pellicle beamsplitter (BP145B4, Thorlabs). The image of the sample is then formed in the plane of the MIR-modified DMD by an achromatic Si/Ge tube lens doublet (AC254-200-E, Thorlabs) with a focal length of 200 mm. The employed DMD (DLP7000BFLP, Texas Instruments) features 1024 $\times$ 768 squared micromirros at a pixel pitch of only 13.68 $\upmu \hbox {m}$, and each of them can be tilted separately to the two distinct angles of $\pm {12}^{\circ }$. Since the standard glass window of commercially available DMDs strongly absorbs wavelength above 2.7 $\upmu \hbox {m}$, the window was replaced in an inert gas atmosphere with a high-transparency MIR CaF_2_ window of 2 mm thickness^20^.

**Figure 2:**
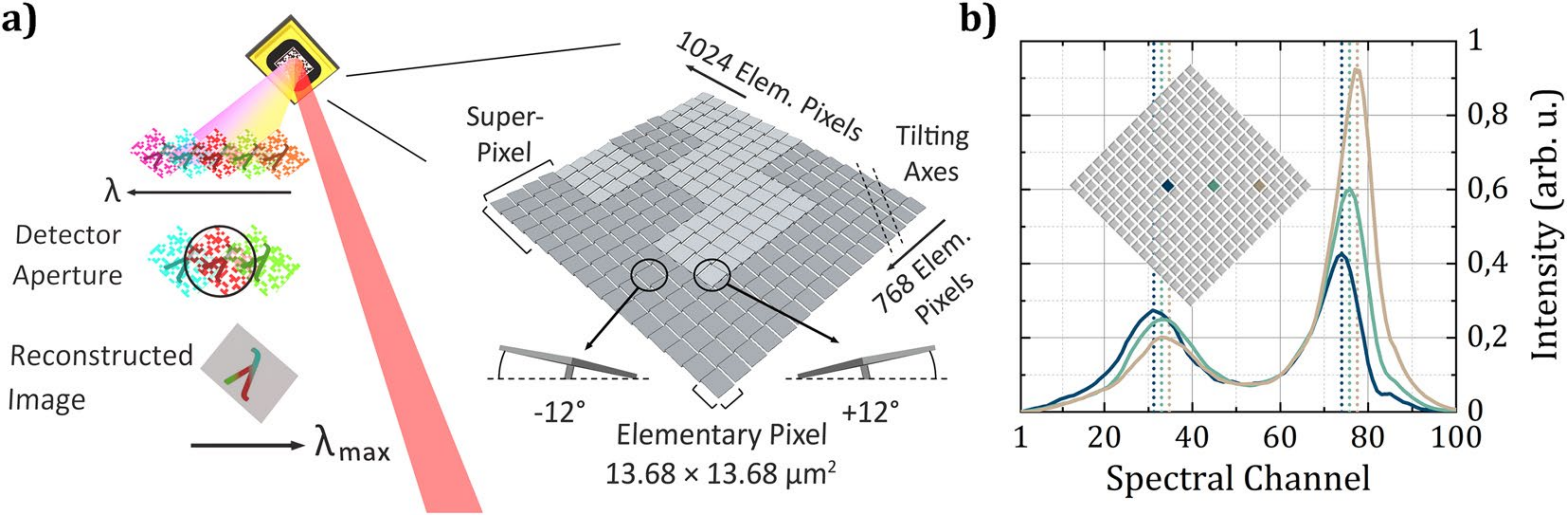
(**a**) Visualization of an exemplary section of the DMD array (right) and principle of operation (left). The DMD spatially masks the captured scene and disperses it due to diffraction at the micromirrors. Overlapping spatially coded images of different wavelengths are at least partially projected on the detector and thus lead to a spectral gradient along the image diagonal. (**b**) Spectral data of three exemplary spatial channels of a hyperspectral 16 $\times$ 16 image resulting from block averaging 64 $\times$ 64 images of a USAF resolution test target recorded at 100 orientations of the rotatable mirror. Depending on the spatial position at the DMD diagonal, wavelengths are pronounced to a different extent.

Directly at the plane of the DMD micromirrors two decisive processes for simultaneous spatial *and* spectral discrimination occur. First, for spatial resolution through SPI, the polychromatic image of the sample is masked according to the orientation of the micromirrors. For this purpose sets of micromirrors (elementary pixels) are grouped to form super-pixels. An applied mask is then composed of a set of e.g. 64 $\times$ 64 super-pixels, which defines the resolution of the reconstructed image (64 $\times$ 64 in this case). Further, the number of micromirrors to form a super-pixel is directly related to the physical size of the latter and thus constrains the spatial resolution. In order to perform SPI, these super-pixels display a time-varying set of binary patterns, where the value 1 corresponds to the deflection state to the left side of the mirror tilting axis—the on-state ($+{12}^{\circ }$). Since this axis is oriented ${45}^{\circ }$ with respect to the mirror matrix, the whole DMD is rotated ${45}^{\circ }$ in plane to provide tilting along a vertical axis and thus simplify alignment. The right section of Fig. 2a illustrates the given keywords by visualizing an exemplary section of the DMD array of 1024 $\times$ 768 elementary pixels. The depicted section shows 4 $\times$ 4 super-pixel, each composed of 4 $\times$ 4 elementary pixels. However, considering the total number of elementary pixels (1024 $\times$ 768), the actual number of elementary pixels forming a super-pixel as well as the total number of super-pixels can be defined by the user, leading to a tunable FoV and tunable spatial resolution.

The second process at the DMD—which enables spectral resolution—is the wavelength dispersion through diffraction at the DMD micromirrors. Detailed reports on the diffraction behavior of DMDs can be found in literature^31–34^. As illustrated in the top section of Fig. 2a, this specific configuration leads to wavelength-dependent masked images that are deflected in different angles according to diffraction—in fact, the DMD thus acts as a spatially modulated grating. The subsequent 4f-system of two spherical 2 inch gold mirrors with focal lengths of 100 mm and 50 mm, respectively, replicates the electric field of the DMD plane without any spatial filtering. It unifies these spectral images in the conjugate plane of the subsequent scanning 1 inch gold mirror. The orientation of this mirror, which is actuated by a motorized rotation stage (RSW60A-T3, Zaber Technologies), then defines the spectral band which is projected onto the ${2}\,\times \, {2}\,{\hbox {mm}^2}$ active area of an MCT single-pixel detector (PCI-4TE-12-2x2, VIGO System) by means of a 1 inch CaF_2_ lens and a ZnSe Objective with focal lengths of 100 mm and 6 mm, respectively.

Figure 2a furthermore indicates a crucial relationship, which links the detector aperture with the spectral resolution of the instrument and the spatial FoV. To detect the entire image projected on the DMD, it is of course necessary to capture all the spatial components deflected from the complete set of super-pixels in the on-state—which might be challenging for a large FoV due to the limited detector aperture. However, particularly for the presented configuration, this is only challenging in one dimension—the one parallel to the mirror tilting axes, in which no relevant diffraction is observed. As indicated in the middle section of Fig. 2a, along the dimension, in which the wavelengths are dispersed, spatially coded images of different wavelengths are overlapping and at least partially detected. Since the entire intensity on the single-pixel detector contributes to the image reconstruction, along this dimension different pixels of the reconstructed image are dominated by different spectral bands. Thus, as illustrated at the bottom section of Fig. 2a, reconstructed images recorded at individual orientations of the rotating mirror feature a spectral gradient in the dimension perpendicular to the mirror tilting axes.

In order to manifest these theoretical considerations, Fig. 2b shows emission spectra extracted from a hyperspectral data cube acquired during a calibration measurement of a USAF resolution test target (R1DS1P, Thorlabs). For this measurement 64 $\times$ 64 images of the highly reflecting chromium elements of the target were acquired for 100 orientations of the rotating mirror leading to 100 spectral channels. As illustrated in the inset, the 64 $\times$ 64 spatial channels were block averaged to 16 $\times$ 16 images. The exemplary spectra depicted are those of three equally spaced spatial channels located at the horizontal DMD diagonal—the actual location is indicated in the inset. All three spectra feature prominent peaks around the spectral channels 30 and 75, which perfectly fit to the emission spectrum of the applied SCL (calibration and a comparison to an FTIR spectrum follow in the next section). However, as a normalization to 0.1 at spectral channel 60 reveals, the peaks are not only shifted, but are also pronounced to different extents. Both observations can be explained by the predicted spectral gradient—the farther to the top right on the reconstructed image, the higher the contribution of longer wavelengths, the farther to the bottom left on the reconstructed image, the higher the contribution of shorter wavelengths. This observation, in turn, leads to two important conclusions. On the one hand, the observed spectral gradient leads to a distortion of the acquired hyperspectral cube—in fact, the resulting skewness turns the cube into a rhombohedron. Thereby, entries in a given spectral channel do not correspond to an identical wavelength band. This distortion, however, can be compensated by a calibration procedure introduced in the following section. On the other hand, due to the fact that a reconstructed image is composed of partial images of different wavelengths, the contribution of specific wavelengths to individual spatial channels can be evaluated by an enhanced calibration method. In fact, by not only using the matrix describing the DMD patterns for reconstruction, but introducing *measured* wavelength dependent calibration matrices acquired with monochromatic illumination, the spectral resolution of the presented instrument can be drastically improved—in principle up to the spectral linewidth of the radiation used for calibration. An experimental demonstration of this advanced calibration method will be the subject of a subsequent publication.

### Reconstruction, calibration and characterization

For hyperspectral data acquisition the developed MIR SPI microscope employs a boxcar integrator (UHFLI, Zurich Instruments) which demodulates the pulsed detector signal before it is sampled by means of a data acquisition board (M3i.4142-Exp, Spectrum). The data acquisition board, the DMD controller board (V-7001, ViALUX) as well as the rotating mirror stage are triggered and controlled by software written in Python which also post-processes the data and reconstructs the acquired hyperspectral images. The applied reconstruction procedure is based on masking the projected image with sequences of a Hadamard matrix consisting of 1 and −1 entries—known as Hadamard sampling^35^. In fact, for 64 $\times$ 64 images the masks (or patterns) applied to the DMD are reshaped rows (1 $\times$ 4096 reshaped to 64 $\times$ 64) of the Hadamard Matrix $\mathbf {H_{4096}}$ (4096 $\times$ 4096). The 1 and −1 entries in the patterns are accounted for by subsequently applying two patterns on the DMD. The first one features super-pixels with 1 entries in the on-state, while the second one features super-pixels with −1 entries in the on-state. In post-processing they are combined to the desired row of the Hadamard matrix by subtracting the subsequent pattern pairs as well as the simultaneously acquired detector signals. Thus, for each row of the Hadamard matrix a *differential* detector signal is acquired, which makes this sampling technique balanced and thus particularly robust against stray light and potential instabilities of the SCL. Finally, the actual images are reconstructed by solving the equation

1 $$\begin{aligned} {\textbf{y}} = \mathbf {H_{4096}} \cdot {\textbf{x}} \end{aligned}$$

with respect to ${\textbf{x}}$. Here, ${\textbf{x}}$ is the desired image projected on the DMD (64 $\times$ 64 reshaped to 4096 $\times$ 1) and ${\textbf{y}}$ is a vector (4096 $\times$ 1) containing the differential detector signals corresponding to the respective patterns (or rows of the Hadamard matrix). Since a Hadamard matrix is up to a factor its own inverse, the image ${\textbf{x}}$ can be reconstructed with a simple dot product between the signals ${\textbf{y}}$ and the Hadamard matrix $\mathbf {H_{4096}}$ without the need for time-consuming matrix inversions. During measurements, the DMD was operated at frame rates of 18.2 kHz leading to display times of 55 $\upmu \hbox {s}$ per pattern. Thus, Hadamard sampled 64 $\times$ 64 images can be acquired in a total of 450 ms (55 $\upmu \hbox {s}$ times 4096 rows of the Hadamard matrix, twice for 1 and -1 entries). The actual reconstruction time strongly depends on the processing power of the employed hardware and is 162 ms for the applied laptop (Precision 7720, Dell). Compressive sampling methods and improved processing power could further reduce acquisition and reconstruction times, respectively.

**Figure 3:**
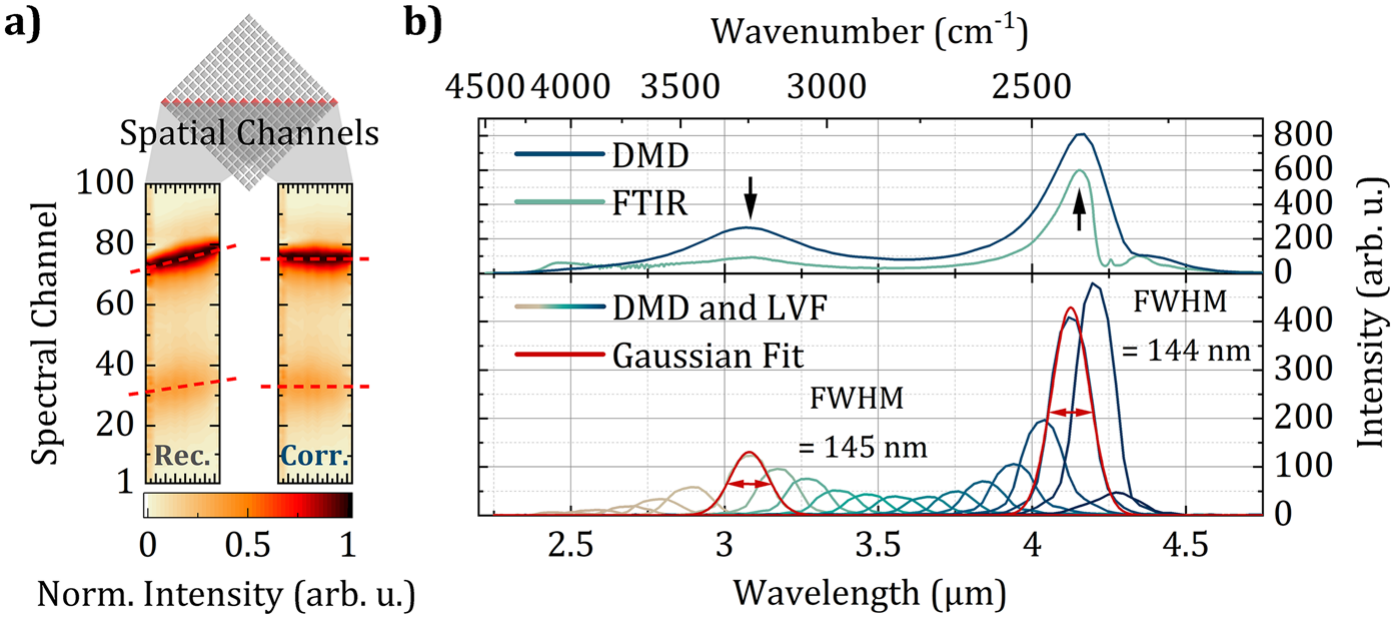
Spectral resolution of the MIR hyperspectral SPI microscope (**a**) False color images of 16 normalized spectra of the spatial channels along the horizontal DMD diagonal (indicated in the mirror matrix on top) resulting from block averaging 64 $\times$ 64 images of a USAF resolution test target along the vertical diagonal. Before (left) and after (right) correction of the spectral gradient, respectively. (**b**) Comparison of the SCL emission spectrum recorded with the developed microscope and an FTIR spectrometer used for wavelength calibration (top). Transmission through a LVF at different positions and Gaussian fits to estimate the spectral resolution of the developed instrument (bottom).

In order to calibrate the spectrum and characterize the resolution of the developed MIR SPI microscope, the USAF resolution test target introduced in the previous section was investigated in detail. At first, 64 $\times$ 64 images were acquired for 100 spectral channels, meaning 100 step-wise scanned orientations of the rotating mirror. For a detailed evaluation of the described spectral gradient, the spatial channels were reduced by block averaging along the vertical DMD diagonal. The resulting 16 spectra represent the spectra along the horizontal DMD diagonal and are illustrated in the left false color image depicted in Fig. 3a. Around the spectral channels 30 and 75 the shift of the SCL emission peaks is clearly visible and indicated by dashed red lines. Interestingly, the observed shift is slightly wavelength dependent, indicating slightly nonlinear dispersion. To correct for this shift—which can be interpreted as a distortion of the hyperspectral cube—the spectral channels were assigned in order to minimize the standard deviation of the peak positions. In numbers, this means the image acquired at rotating mirror position 75 is not anymore assigned to spectral channel 75, rather only the central axis along the mirror tilting axes is assigned to this specific spectral channel. Depending on the wavelength, in the directions left and right to the center the assignment is subsequently incremented and decremented, respectively. The right side of Fig. 3a shows a false color image of the 16 spectra resulting from this correction procedure. The observed shift of the measured peaks is no longer present. Thus, as desired for hyperspectral cubes, certain spectral channels now feature quasi-monochromatic spatial images.

To calibrate the wavelengths, a global spectrum was extracted by averaging the 4096 spatial channels in each spectral channel. Since the chromium elements of the USAF resolution test target feature an essentially flat reflection spectrum in the accessible spectral range, the resulting spectrum—illustrated in the top section of Fig. 3b—is in good agreement with a reference spectrum of the applied SCL acquired with a commercial FTIR spectrometer (VERTEX 70, Bruker). The two prominent intensity peaks of the SCL at 3.08 $\upmu \hbox {m}$ and 4.16 $\upmu \hbox {m}$ (indicated by black arrows) were used for wavelength calibration using linear interpolation.

In an additional experiment similar hyperspectral cubes (64 $\times$ 64 $\times$ 100) were recorded while a linear variable filter (LVF-2.5-5.0-3.5-15-0.5, Vortex Optical Coatings) covering the 2.5 $\upmu \hbox {m}$ to 5 $\upmu \hbox {m}$ range was sequentially moved across the beam path. Again, the spatial channels were averaged to reveal the spectra depicted in the lower section of Fig. 3b. The fact that each position of the LVF resulted in bell-shaped spectra without any side maxima indicates that there is no overlap with higher diffraction orders. A Gaussian fit of the spectra measured near the SCL intensity peaks at center wavelengths of 3.08 $\upmu \hbox {m}$ and 4.12 $\upmu \hbox {m}$ (due to strong CO_2_ absorption centered at around 4.3 $\upmu \hbox {m}$, a shorter wavelength was used) revealed a FWHM of 145 nm and 144 nm, respectively. According to the LVF manufacturer, at these center wavelengths, the inserted filters feature transmission spectra with a FWHM of 61.6 nm and 82.4 nm, respectively. Theoretically, the measured spectra result from the convolution of the microscope’s instrumental response function with the input spectrum, i.e. the transmission spectra of the LVF. Due to different physical broadening processes, such functions can often be estimated as convolutions of Gaussian and Lorentzian functions^36^. However, the assumption of essentially Gaussian functions considerably simplifies our estimate of the spectral resolution, since the convolution of two Gaussian functions is again Gaussian and the respective variances add up. Additionally, for a Gaussian distribution, the FWHM is proportional to the standard deviation. Thus, the ${FWHM_{inst}}$ of our instrumental response function at a wavelength of 4.12 $\upmu \hbox {m}$—which provides a measure for the lower limit of the spectral resolution $\delta _\lambda$—can be estimated by

2 $$\begin{aligned} \delta _\lambda \le {FWHM_{inst}} \approx {\sqrt{FWHM_{meas}^2 - FWHM_{LVF}^2}} = 118.1\,{\hbox {nm}} , \end{aligned}$$

where ${FWHM_{meas}}$ and ${FWHM_{LVF}}$ relate to the measured spectra and the transmission spectra of the LVF, respectively. Although an approach to further enhance the spectral resolution was discussed in the previous section, measurements of real samples in the following section manifest the sufficiency of this spectral resolution for chemical investigations.

**Figure 4:**
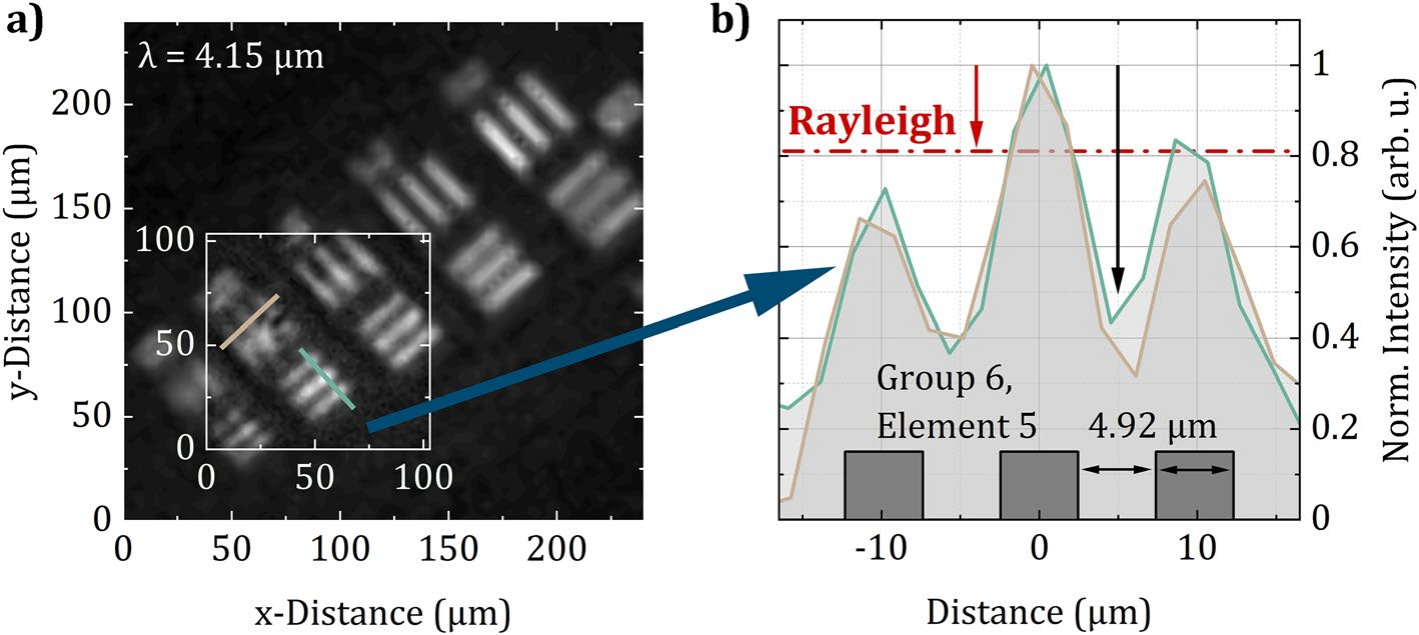
Spatial resolution of the MIR hyperspectral SPI microscope. (**a**) Collage of two 64 $\times$ 64 images of the elements 2–6 of group 6 of a USAF resolution test target acquired with super-pixels consisting of 7 $\times$ 7 and 3 $\times$ 3 micromirrors leading to FoVs of 240 $\times$  240 $\upmu \hbox {m}^2$ and 103 $\times$  103 $\upmu \hbox {m}^2$, respectively. (**b**) Profiles of element 5 of group 6 of a USAF resolution test target acquired with the MIR SPI microscope verifying diffraction-limited spatial resolution.

In order to verify the diffraction-limited spatial resolution, Fig. 4a shows images of the elements 2–6 of group 6 of the USAF resolution test target acquired at a wavelength $\lambda$ of 4.15 $\upmu \hbox {m}$. In fact, the illustrated image is a collage of two 64 $\times$ 64 images acquired with different sizes of super-pixels leading to different FoVs. While the larger image was recorded with super-pixels composed of 7 $\times$ 7 micromirrors resulting in a squared FoV of about 240 $\times$  240 $\upmu \hbox {m}^2$, the smaller high-resolution image was recorded with super-pixels composed of 3 $\times$ 3 micromirrors resulting in a FoV of about 103 $\times$  103 $\upmu \hbox {m}^2$. Since both images were acquired in 450 ms, this comparison perfectly emphasizes the great flexibility of SPI microscopy considering a tunable FoV and tunable spatial resolution—high resolution images of areas of interest as well as large overview screenings can be recorded at exceptional acquisition times. The actual FoV dimensions were calculated by evaluating the profiles of element 5 of group 6 of the test target depicted in Fig. 4b. The linewidth of this element is 4.92 $\upmu \hbox {m}$ leading to twice this distance between two intensity peaks in the illustrated image. In addition, since a 64 $\times$ 64 image of 7 $\times$ 7 micromirrors per super-pixel leads to an active area on the DMD of 448 $\times$ 448 micromirrors which is about ${6.13}\,\times \,{6.13}\,{\hbox {mm}^2}$, this calculation is in good agreement with the 25x magnification of the objective. As indicated by the arrows in Fig. 4b the intensity drop between two peaks of the normalized profiles exceeds even 50% and thus easily satisfies the Rayleigh criterion. Furthermore, according to the Abbe diffraction limit, the smallest resolvable structure $d = \frac{\lambda }{2 NA} = \frac{{4.15}\,{\upmu \hbox {m}}}{0.8} = {5.19}\,{\upmu \hbox {m}}$, which is not only closest to the linewidth of element 5 of group 6 compared to all other elements on the USAF resolution test target, but also marginally larger, which is why we consider the developed SPI microscope to be diffraction-limited.

### Hyperspectral imaging

#### Human blood cells

**Figure 5:**
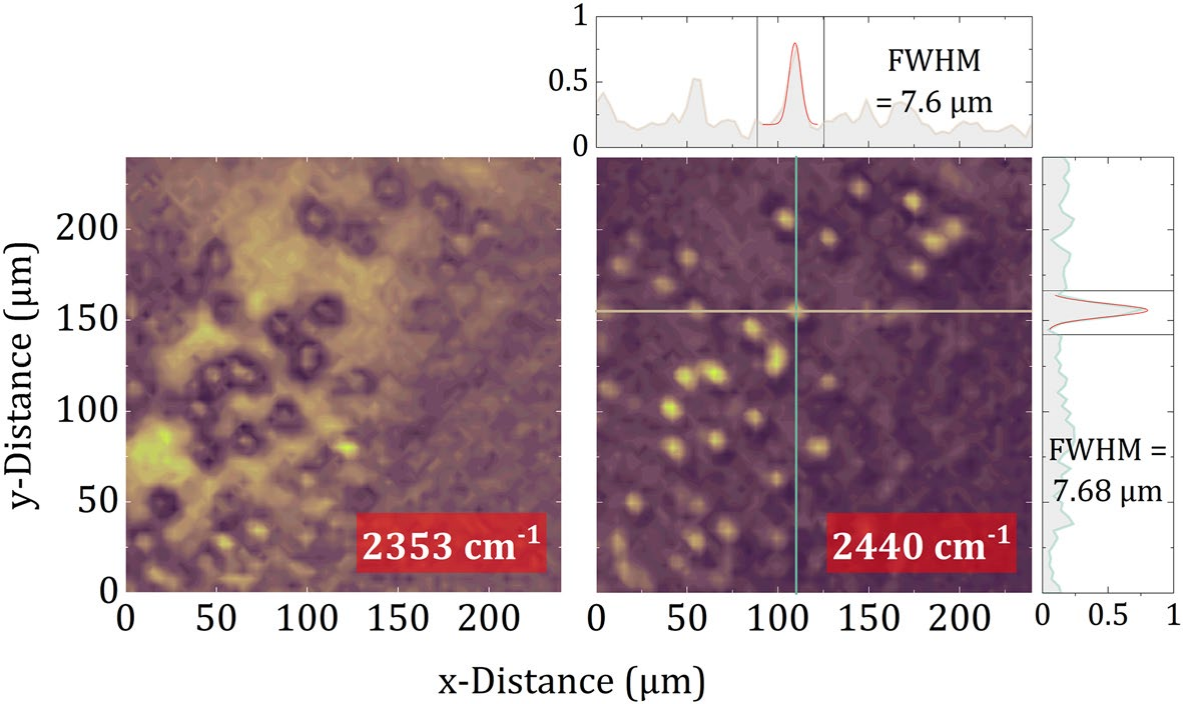
MIR SPI microscope images of human red blood cells. Spectral images at two exemplary wavenumbers (2353 $\hbox {cm}^{-1}$ and 2440 $\hbox {cm}^{-1}$) have been extracted from the acquired hyperspectral data cube and plotted as normalized intensity images. The indicated profiles through an exemplary blood cell reveal a FWHM of about 7.6 $\upmu \hbox {m}$, which is in agreement with the expected cell diameter.

To illustrate the capabilities of the developed MIR SPI microscope, Fig. 5 shows a set of normalized intensity images of a human blood smear on an aluminum mirror recorded at different wavelengths. The depicted 64 $\times$ 64 images were acquired at two different orientations of the rotating mirror in 450 ms each. The chosen orientations led to images at wavenumbers of 2353 $\hbox {cm}^{-1}$ and 2440 $\hbox {cm}^{-1}$. Both images clearly reveal the presence of red blood cells. Since such cells are disk-shaped with an expected diameter of 6.2 $\upmu \hbox {m}$ to 8.2 $\upmu \hbox {m}$, they represent an ideal biological sample to examine the diffraction-limited spatial resolution and thus served as a real-life resolution test target^37^. However, while the image at 2440 $\hbox {cm}^{-1}$ shows circular cells, the same cells appear ring-shaped in the image at 2353 $\hbox {cm}^{-1}$, indicating slight chromatic aberration. An evaluation with Gaussian fits of the profiles through an exemplary particle in the 2440 $\hbox {cm}^{-1}$ image reveals a FWHM of about 7.6 $\upmu \hbox {m}$ which is in excellent agreement with the expected cell diameter. Thus, this measurement emphasizes the capability of *diffraction-limited* MIR microscopy of biological and biomedical samples with acquisition times in the tens of millisecond time regime.

#### Polymer multilayers

**Figure 6:**
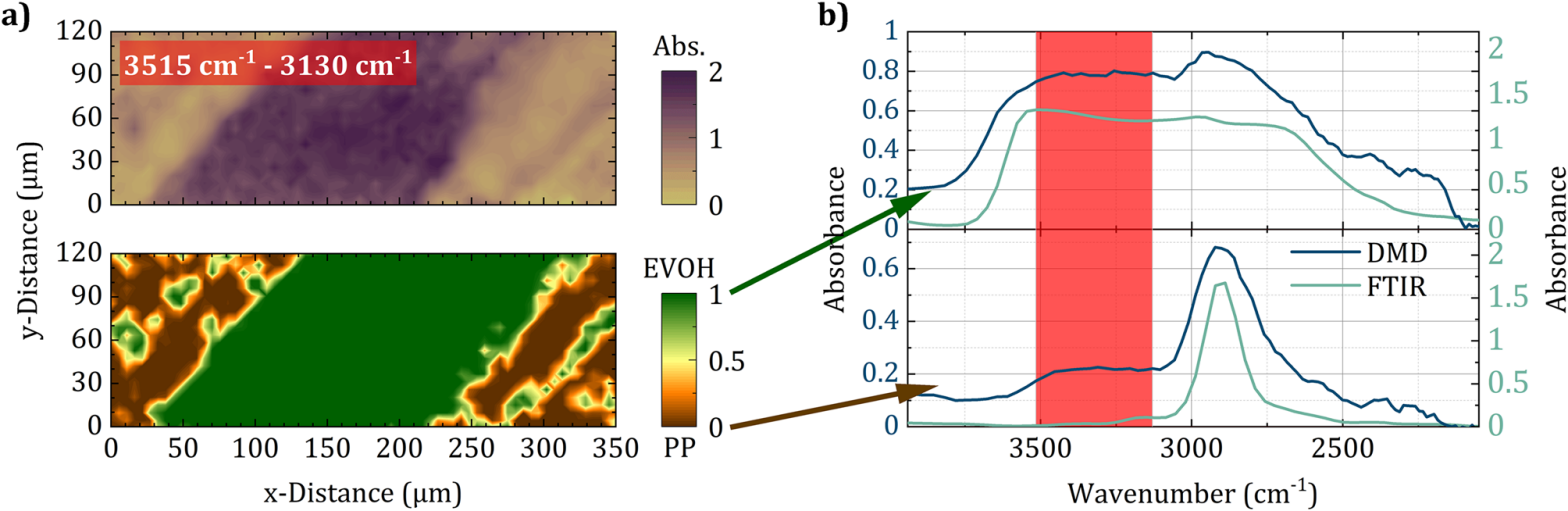
(**a**) Absorbance image of PP/EVOH/PP multilayer film calculated by averaging the spectral band from 3515 $\hbox {cm}^{-1}$ to 3130 $\hbox {cm}^{-1}$ (OH stretching bond, top), and probability estimates for EVOH and PP calculated by means of logistic regression (bottom). (**b**) Global EVOH and PP spectra (blue) resulting from weighing the spectra in each spatial channel according to the respective probability, and a comparison to conventional FTIR spectra of pure EVOH and PP (turquoise) also recorded in reflection configuration. The characteristic absorption band used for averaging in the top section of (a) is indicated (red).

Figure 6 illustrates hyperspectral data of a microtome section of a polypropylene/ethylene vinyl alcohol/polypropylene (PP/EVOH/PP) multilayer film acquired with the presented microscope. The presented data thus represent, to the best of our knowledge, the first chemical images recorded with an SPI approach in the MIR. EVOH is a flexible oxygen barrier material commonly used in shelf-stable food packaging where oxygen degrades the quality of products and shortens their shelf life. Insight into the chemical composition of multilayer packaging materials—where EVOH is often implemented as an intermediate layer—is of great importance for smart product design. Therefore, the top section of Fig. 6a depicts an absorbance image of the microtome cross-section of a PP/EVOH/PP multilayer film on an aluminum mirror as substrate. The absorbance $\textrm{A}$ is calculated according to Beer’s law by

3 $$\begin{aligned} \textrm{A} = -\log _{10}\left( \frac{\mathrm {I_{s}}}{\mathrm {I_{ref}}}\right) \text {,} \end{aligned}$$

where $\mathrm {I_{s}}$ and $\mathrm {I_{ref}}$ are intensities recorded during a sample measurement and a reference measurement, respectively^38,39^. Thus, for this measurement two hyperspectral cubes with 64 $\times$ 64 spatial channels in each of the 100 spectral channels were acquired—one for the sample measurement ($\mathrm {I_s}$) and one for a reference measurement of the plain aluminum mirror ($\mathrm {I_{ref}}$). The acquisition of such a hyperspectral cube with a total of 4$\times 10^{5}$ channels takes about 50 s. To avoid destroying the sample, during this experiment the illumination was dimmed with a 50% neutral density filter (NDIR03A, Thorlabs). The depicted hyperspectral absorbance data is calculated from the two cubes according to Eq. (3). For better visualization the spectral band from 3515 $\hbox {cm}^{-1}$ to 3130 $\hbox {cm}^{-1}$ was averaged—in this range, in contrast to PP, EVOH is expected to feature strong absorption (additional wavelengths are shwon in supplementary file 1). Additionally, the FoV was cropped to highlight the most characteristic spatial features. Already in this absorbance image, a layered structure with high absorption in the center is clearly visible and indicates a strong presence of EVOH. However, by evaluating the whole hyperspectral cube by means of the mathematical method of logistic regression, comprehensive chemical classification is feasible^40^. By using sections of the different absorption regions to classify EVOH and PP, the applied logistic regression model provides the probability estimates depicted in the lower section of Fig. 6a, which reveal the spatial distribution of EVOH and PP—even small inclusions of EVOH within the PP layers are traceable. Furthermore, the global EVOH and PP spectra depicted as blue graphs in Fig. 6b result from weighing the spectra in each spatial channel according to the respective probability provided by the logistic regression. Despite the limited spectral resolution, the EVOH and PP spectra clearly differ, which is the basis for successful chemical analysis. The turquoise graphs show a comparison with absorption spectra of pure EVOH and PP films recorded with a conventional FTIR microscope (LUMOS, Buker) in reflection configuration at a spectral resolution of 64 $\hbox {cm}^{-1}$—which is comparable to the estimated spectral resolution of the developed instrument at least for the longer wavelengths in the accessible spectral range. Since both measurements feature the same absorption bands at similar spectral resolution, the developed hyperspectral MIR SPI microscope enables non-destructive and label-free chemical imaging without cost-intensive detector arrays and spectrometers at acquisition times below 1 minute for a total of $4\times 10^{5}$ spatial and spectral channels.

## Discussion

An approach to MIR hyperspectral imaging microscopy that *does not* require an expensive array detector or a dedicated spectrometer, was presented. The method is based on the principles of SPI and additionally exploits the diffraction of MIR radiation at the micromirrors of a MIR-modified DMD. Thus, it enables a *single* DMD to operate as hyperspectral imager in the MIR with acquisition times in the tens of millisecond time regime.

A detailed characterisation of the developed MIR SPI microscope, including a suitable calibration method, was presented. *Diffraction-limited* spatial resolution was experimentally validated by means of a USAF resolution test target. A native spectral resolution better than 118.1 nm was estimated using a MIR LVF. Further, an approach for drastic improvement of the spectral resolution was presented. The sufficiency of the realized specifications for chemical imaging was demonstrated by the investigation of a human blood smear and a microtome cross-section of a PP/EVOH/PP multilayer film. The diffraction-limited spatial resolution allowed us to image individual red blood cells within the smear. The acquired hyperspectral data of the multilayer polymer film represent, to the best of our knowledge, the first chemical images recorded with an SPI approach in the MIR. The spatial distribution of EVOH and PP was evaluated by means of logistic regression and the calculated spectra were compared to spectra of the pure polymers acquired with a conventional FTIR microscope ensuring proper chemical classification. The acquisition time of 450 ms for quasi-monochromatic 64 $\times$ 64 images and about 50 s for hyperspectral cubes with 64 $\times$ 64 $\times$ 100 channels outperforms conventional scanning FTIR microscopy by orders of magnitude. Thus, the developed hyperspectral imager drastically improves the sample throughput in MIR chemical and biomedical imaging—especially considering a new degree of flexibility with *tunable FoV* and *tunable spatial resolution*, which directly accounts for the effective acquisition time. However, the presented approach not only designates a fast and cost-effective improvement for hyperspectral MIR microscopy. The method of applying a MIR-modified DMD as a *single* element for simultaneous spatial and spectral sensing can be easily transferred to numerous disciplines. The presented approach can replace conventional hyperspectral IR cameras based on array detectors and spectrometers, e.g. in remote sensing, in-line process monitoring, leak detection and many other areas.

## Methods

The human blood smear sample consists of an air-dried human blood smear on an aluminum mirror. Human whole blood samples were obtained from the blood donor center of the Austrian Red Cross in Vienna. Aliquotes of 10 ml whole blood were taken from diagnostic samples of two anonymized blood donors after completion of the routine diagnostic, which follows blood donation.

The PP/EVOH/PP multilayer sample is a microtome cross-section with about 20 $\upmu \hbox {m}$ thickness fixed on a reflective aluminum mirror. The total film thickness is about 1150 $\upmu \hbox {m}$, the EVOH intermediate layer has a varying thickness of 60 $\upmu \hbox {m}$–120 $\upmu \hbox {m}$ and is centered between two PP layers of equal dimension.

## Supplementary Information

Supplementary Information.

## Data Availability

The datasets used and analyzed during the current study are available from the corresponding author upon reasonable request.
