# Supplementary figures and images for: Diffraction-limited hyperspectral mid-infrared single-pixel microscopy
Source: Sci Rep. 2023 Jan 6;13:281. doi: 10.1038/s41598-022-26718-6 (PMC9822906; doi:10.1038/s41598-022-26718-6)

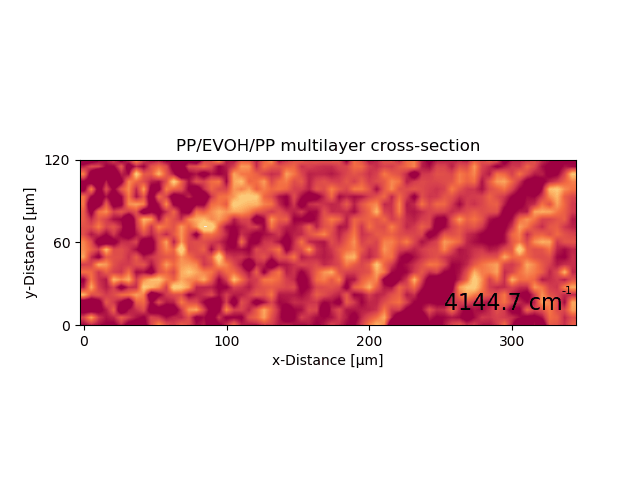

Supplement: Supplementary file 1 — Supplementary Information. [file 41598_2022_26718_MOESM1_ESM.gif]
